# Supplementary material for: Analysis of the different interventions scenario for programmatic measles control in Bangladesh: A modelling study
Source: PLoS One. 2023 Jun 29;18(6):e0283082. doi: 10.1371/journal.pone.0283082 (PMC10310053; doi:10.1371/journal.pone.0283082)
Supplement: Attachment — Submitted filename: Response to reviewer comments.docx [file pone.0283082.s002.docx]

Response to reviewer: Analysis of the different interventions scenario and cost-effective strategy for programmatic measles control in Bangladesh: a modelling study

Md Abdul Kuddus^1, 2*^, Azizur Rahman^3^, Farzana Alam^4^ and M. Mohiuddin^5^

^1^Australian Institute of Tropical Health and Medicine, James Cook University, Townsville, QLD, Australia

^2^Department of Mathematics, University of Rajshahi, Rajshahi-6205, Bangladesh

^3^School of Computing and Mathematics, Charles Sturt University, Wagga Wagga, NSW 2678, Australia

^4^Department of Electrical and Electronic Engineering, Rajshahi University of Engineering & Technology, Rajshahi-6204, Bangladesh

^5^Department of Mathematics, Comilla University, Cumilla-3506, Bangladesh

We thank the reviewer for their assessment of our study, and the comments below. We have made changes to the manuscript and replied to other comments below. Reviewer comments are given in black, with our replies in red.

#################################################################################

Reviewer #1: This is a very well written scientific work. It puts together the different arrangements that can either be given individually or in combination. This piece of work would further strengthen if the cost benefit analysis as well as it will be used as a strong advocacy tool.

Response: Thank you for your valuable comment. We have now added cost benefit analysis of different intervention strategies including distancing, vaccination and treatment to the revised manuscript. Please see page 17-23, line 368 – 521 and page 26-27, line 610-637.

##################################################################################

Reviewer #2: This is an well written article on the topic of measles in Bangladesh. The mathematical models presented in the paper are really interesting. I would leave a minor comment on enriching the discussion with few other similar studies and citing them. You have already discussed your findings in the discussion, it would be better to have some more similar examples from other LMICs.

Response: We thank the reviewer for this wonderful suggestion. We have cited some other similar studies in LMICs in the relevant section as well as discussion section to the revised manuscript. Please see page 22-24, line 481 – 488, 527 – 538 and page 26-27, line 595 – 600, 624 – 630.
